# Copper-coated carbon nanotube surfaces for inhibiting biofilm formation by *Staphylococcus aureus* and *Pseudomonas aeruginosa*

**DOI:** 10.1016/j.tcsw.2026.100176

**Published:** 2026-06-08

**Authors:** Abiraami Kalaiselvi Marimuthu, Rithik Roshini Gopi, Jahnavi Padmavathi Sridhar, Kalaivani Ravi, Aditi Roy, Sudha Ramaiah, Anand Anbarasu

**Affiliations:** a Department of Biomedical Sciences, School of Biosciences and Technology (SBST), Vellore Institute of Technology (VIT), Vellore 632014, Tamil Nadu, India; b Department of Biotechnology, SBST, VIT, Vellore 632014, Tamil Nadu, India; c Medical and Biological Computing Laboratory, SBST, VIT, Vellore 632014, Tamil Nadu, India; d Department of Biosciences, SBST, VIT, Vellore 632014, Tamil Nadu, India

**Keywords:** Antimicrobial, Extracellular polymeric substances, Nanotechnology, Nanotube, Surface modification

## Abstract

Biofilm-associated infections caused by clinically important pathogens such as *Staphylococcus aureus* (*S. aureus*) and *Pseudomonas aeruginosa* (*P. aeruginosa*) remain a major challenge in healthcare settings due to enhanced antimicrobial resistance and persistence. In recent years, copper-coated carbon nanotubes (Cu-CNTs) have gained considerable attention as promising antimicrobial nanomaterials for preventing biofilm formation on medical devices and hospital-associated surfaces. This review summarizes recent advances in the development and application of Cu-CNT-based antimicrobial coatings, with an emphasis on their antibacterial and antibiofilm activities against Gram-positive and Gram-negative pathogens. The synergistic combination of the high surface-area-to-volume ratio of CNTs and the potent antimicrobial properties of copper (Cu) ions enhances microbial inhibition. Previous studies suggest that Cu-CNTs interfere with initial bacterial adhesion, inhibit biofilm maturation, and disrupt established biofilms through mechanisms involving oxidative stress generation, membrane destabilization, and cellular damage. Furthermore, the review discusses the physicochemical characteristics, antimicrobial mechanisms, biomedical applications, and potential challenges associated with Cu-CNT coatings, including toxicity and biocompatibility concerns. Overall, Cu-CNT-based coatings represent a promising strategy for developing durable antimicrobial surfaces to control chronic biofilm-associated infections and reduce healthcare-associated contamination.

## Highlights

• Cu-CNTs show strong antibiofilm activity against *Staphylococcus aureus* and *Pseudomonas aeruginosa.*
• Significantly reduce bacterial adhesion and biofilm formation.
• Act via oxidative stress and membrane disruption.
• Effective against both Gram-positive and Gram-negative bacteria.
• Low toxicity with potential for biomedical coatings.

## 1 Introduction

Biofilm-associated infections pose a major challenge in both clinical and industrial settings due to their persistence and increased resistance to conventional antimicrobial therapies. Clinically significant pathogens, including *P. aeruginosa*, *S. aureus*, and *Klebsiella pneumoniae* (*K. pneumoniae*), possess a strong ability to form biofilms on the surfaces of medical devices such as catheters, implants, and ventilators. These biofilm-mediated infections are often chronic, recurrent, and difficult to eradicate, particularly in immunocompromised individuals, leading to severe healthcare complications. The enhanced antibiotic resistance is attributed to multiple factors, including limited antibiotic penetration, altered physicochemical conditions within the biofilm microenvironment, metabolic heterogeneity, and the presence of persister cells. Therefore, standard antimicrobial treatments are often ineffective, underscoring the urgent need to develop alternative, effective approaches to prevent and control biofilm formation (Tong et al., 2015).

In recent years, nanotechnology has emerged as a promising strategy for the prevention and management of microbial infections, particularly those associated with biofilm formation. Nanomaterials have distinct physicochemical properties, including a high surface area-to-volume ratio, enhanced responsiveness, and the ability to interact closely with microorganisms. Various nanomaterials, including metal and metal oxide nanoparticles, have attracted considerable attention for biomedical and antimicrobial applications owing to their unique physicochemical and redox properties (Nag et al., 2024). Among these, CNTs have attracted considerable attention for their high durability, electron-conducting properties, and antimicrobial activity. CNTs interact with bacterial membranes, causing membrane ruptures and cellular damage, thereby enhancing their antibacterial activity.

In addition to the well-established antimicrobial properties of CNTs, incorporating metal nanoparticles has further enhanced their therapeutic potential. Among various metals, Cu has emerged as a widely studied antimicrobial agent due to its broad-spectrum activity against bacteria, fungi, and viruses. The antimicrobial activity of Cu is mediated through multiple mechanisms, including the generation of reactive oxygen species (ROS), disruption of cellular membranes, protein denaturation and misfolding, and inhibition of nucleic acid synthesis and DNA replication. Furthermore, Cu ions interfere with essential enzymatic and metabolic processes within microbial cells, ultimately leading to oxidative damage, cellular dysfunction, and microbial cell death (Scott, 2013).

The integration of Cu with CNTs yields a multifunctional nanocomposite with synergistic antimicrobial properties. Cu-CNTs combine the unique structural, mechanical, and physicochemical characteristics of CNTs with the potent broad-spectrum antimicrobial activity of Cu. This synergistic interaction enhances the contact efficiency between the nanomaterials and microbial cells, thereby improving the inhibition of bacterial adhesion and subsequent biofilm formation. Studies have demonstrated that Cu-CNTs effectively interfere with the initial attachment of bacterial cells to surfaces, suppress biofilm maturation, and facilitate the disruption of pre-established biofilms. These antibiofilm effects are primarily associated with enhanced membrane interactions, oxidative stress induction, and structural destabilization of microbial communities (Giunchedi et al., 2020).

Furthermore, Cu-CNT-based nanocomposites offer several advantages over conventional antimicrobial agents, including lower toxicity, prolonged antimicrobial efficacy, and advanced applications for coating medical devices. These properties highlight the potential of Cu-CNTs as promising candidates for combating the growing global burden of biofilm-associated infections and antimicrobial resistance. The synergistic integration of Cu and CNTs enhances antibiofilm activity through combined physical disruption of microbial membranes and oxidative stress–induced antimicrobial effects. Thus, Cu-CNT surfaces have demonstrated greater effectiveness than uncoated carbon nanotube surfaces in inhibiting mixed microbial populations and biofilm formation involving *S. aureus* and *P. aeruginosa*. Understanding the synergistic interactions between Cu and carbon nanotube surfaces may facilitate the development of novel antimicrobial coatings for medical devices and healthcare-associated applications (Donaldson et al., 2006).

## 2 Biofilm formation and its stages

A biofilm is a highly organized and structured microbial community that adheres to biotic or abiotic surfaces and is embedded within a self-produced extracellular polymeric substance (EPS) matrix. The EPS matrix provides structural integrity, facilitates irreversible surface attachment, and protects microbial cells from environmental stress, host immune responses, and antimicrobial agents, thereby enhancing microbial survival and persistence under adverse conditions (Costerton et al., 1999).

The biofilm formation begins with the attachment of bacteria, in this case *S. aureus* and *P. aeruginosa*, to the surface. This initial attachment is reversible, as microbial cells adhere to the surface through weak physicochemical interactions, including van der Waals forces and electrostatic interactions. However, they attach with their pili, fimbriae, and flagella. This activates intracellular signaling pathways, particularly those regulated by cyclic diguanylate monophosphate (*c*-di-GMP), a key secondary messenger that promotes the transition from a motile to a sessile lifestyle and stimulates EPS production (Bjarnsholt et al., 2013).

The EPS matrix consists of polysaccharides, proteins, lipids, and extracellular DNA; together, these components form a scaffold that protects bacterial cells from antibiotics, dehydration, and various environmental stresses. Mature biofilms often form mushroom- or tower-like three-dimensional structures. The multilayered organization restricts the diffusion of oxygen and nutrients to deeper biofilm layers, leading to reduced metabolic activity, slow growth, or dormancy, which contribute to increased antimicrobial tolerance (Flemming and Wingender, 2010).

Biofilms are associated with numerous chronic and persistent infections, including chronic wound infections, implant-associated infections, and postoperative bone infections. Bacteria residing in the deeper layers of the biofilm exhibit enhanced tolerance to antibiotics and disinfectants due to the protective EPS matrix. The EPS matrix acts as a physical barrier that limits the penetration of antimicrobial agents and promotes the development of metabolically inactive or dormant bacterial populations. Consequently, many conventional antibiotics exhibit reduced efficacy against biofilms because they primarily target actively growing cells. For example, *β*-lactam antibiotics inhibit peptidoglycan synthesis during cell division and are therefore less effective against dormant bacteria embedded within mature biofilms, often leading to treatment failure and persistent infections. *S. aureus* and *P. aeruginosa* are among the predominant biofilm-forming pathogens exhibiting a strong capacity to establish robust and persistent biofilms (Donlan, 2002; Kulandaivelu et al., 2026). A wide range of biofilm-forming microorganisms are associated with infections in distinct anatomical sites, highlighting the broad clinical impact of biofilm-mediated infections (Table 1).

**Table 1:** Organisms responsible for biofilm formation in different sites.

Location in human body	Common bacteria	Types of infection	References
Oral cavity	*Streptococcus mutans*, *Streptococcus sanguinis*	Dental caries Dental plaque	(Chambers, 2001)
Tonsils	*Streptococcus pycogens*, *S. aureus*	Tonsillitis	(Costerton et al., 1999)
Lungs	*P. aeruginosa*, *S. aureus*, *Haemophilus influenzae* (*H. influenzae*)	Chronic lung infections	(Hall-Stoodley et al., 2004)
Nasal cavity	*S. aureus*	Sinusitis	(Uchida et al., 2010)
Middle ear	*H. influenzae,* *Streptococcus pneumoniae*	Otitis media	(Hatt and Rather, 2008)
Urinary tract	*Escherichia coli* (*E. coli*), *K. pneumoniae*	Urinary tract infection	(Flores-Mireles et al., 2015)
Skin and wound	*S. aureus*, *P. aeruginosa*	Chronic wound infection	(Vacca, 2017)
Blood stream	*Staphylococcus epidermidis (S. epidermidis)*, *S. aureus*	Sepsis	(Cavinato et al., 2020)
Heart valves	*S. aureus*, *Streptococcus viridans*	Infective endocarditis	(Chambers, 2001)
Gastrointestinal tract	*Helicobacter pylori*, *E. coli*,	Ulcer, intestinal infections	
Medical implants	*S. epidermidis,* *S. aureus*	Implant-associated infection	(Hall-Stoodley et al., 2004)

Biofilm formation is a highly regulated multistep process that progresses through four major stages:

• Initial attachment
• Irreversible adhesion
• Maturation
• Dispersion

### 2.1 Initial attachment

Initial attachment is one of the most critical stages of biofilm formation, during which planktonic bacterial cells encounter and adhere to a surface. The initial attachment stage is typically reversible, with bacterial cells interacting transiently with surfaces via van der Waals, electrostatic, and hydrophobic forces, allowing cells to intermittently attach to and detach from the surface. Although electrostatic repulsion may exist between the bacterial cell and the substrate, surface appendages such as fimbriae, pili, and flagella facilitate close surface contact and help overcome these repulsive forces, thereby promoting attachment. Thereafter, they would slowly form a layer of organic molecules on the surface that influences bacterial adhesion by modifying it. If the attachment is stable, the bacteria begin releasing extracellular polymeric substances (EPS), which strengthen the interaction and lead to irreversible attachment (O'Toole et al., 2000).

### 2.2 Irreversible attachment

The transition from reversible attachment to stable surface colonization marks a crucial stage in biofilm development and is facilitated by EPS production. The EPS matrix, primarily composed of polysaccharides, proteins, and extracellular DNA (eDNA), provides structural integrity and reinforces both cell–surface and cell–cell interactions. Furthermore, it supports the genetic and phenotypic adaptations necessary for biofilm establishment. Following surface attachment, cyclic di-GMP-dependent signaling pathways are activated, promoting the expression of genes involved in biofilm formation and maintenance. During this phase, bacterial cells proliferate and aggregate into microcolonies that progressively expand and merge, ultimately forming a mature biofilm (Donlan, 2002).

### 2.3 Maturation

The maturation stage is characterized by extensive bacterial proliferation and differentiation, leading to the development of complex three-dimensional biofilm architectures, including mushroom-like and tower-like structures. During maturation, extensive production of the EPS matrix occurs, forming a protective barrier that enhances structural stability, facilitates nutrient retention, and protects embedded bacterial cells from antibiotics, immune defenses, and adverse environmental conditions. Also, they help with nutrient uptake, oxygen exchange, and waste release. The mature biofilm would exhibit physiological heterogeneity, with cells in different regions showing varying stages of metabolic activity. This stage constitutes the most advanced phase of biofilm development, during which the biofilm attains a highly organized three-dimensional architecture and exhibits enhanced resistance to antimicrobial agents and host immune defenses (Di Martino, 2018).

### 2.4 Dispersion

**Fig. 1:**
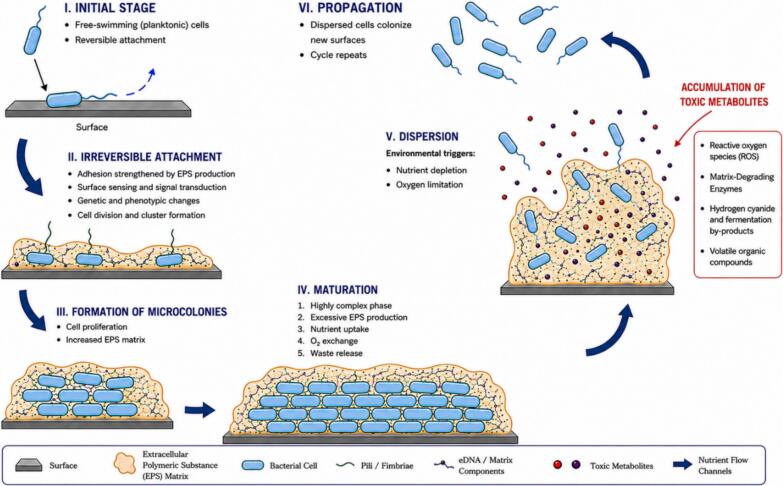
Different stages of Biofilm formation.

Dispersion is the final stage of the biofilm life cycle, during which bacterial cells are released from the mature biofilm, enabling colonization of new surfaces and initiating subsequent biofilm formation. This process is often triggered by environmental stresses, including nutrient depletion, oxygen limitation, and the accumulation of toxic metabolic by-products (Fig. 1). In response to these conditions, biofilm-associated cells produce matrix-degrading enzymes, such as DNases and proteases, which degrade components of the extracellular polymeric substance (EPS) matrix. The degradation of the EPS framework compromises biofilm integrity, thereby facilitating the detachment and dissemination of bacterial cells onto adjacent surfaces and into surrounding environments (Guilhen et al., 2017).

## 3 Carbon nanotube-based antibiofilm materials

CNT-based material has shown significant antimicrobial and anti-adhesive properties. Previous studies reported that the CNT surface exhibits strong antimicrobial activity and inhibits biofilm formation. Biofilm formation is a major problem in most healthcare settings due to its resistance to antibiotics and the host's response to the disease. This problem is overcome by using carbon nanotubes rather than conventional materials. A CNT is a cylindrical nanostructure composed of rolled graphene sheets and can exist as either a single-walled carbon nanotube (SWCNT) or a multi-walled carbon nanotube (MWCNT). Their nanoscale dimensions confer unique physicochemical properties, including a high surface-area-to-volume ratio, exceptional mechanical strength, and excellent electrical conductivity. Numerous studies have reported that CNT-based surfaces effectively reduce biofilm formation by both Gram-positive and Gram-negative bacteria, highlighting their potential for biomedical and antimicrobial applications (Ahmed et al., 2012; Bowden et al., 2025; Bowden et al., 2023; Gomes et al., 2022).

CNTs possess a needle-like structure that can damage bacterial cells upon direct contact. When bacteria attempt to attach to a CNT-coated surface, the sharp nanotubes can penetrate the cell membrane, causing physical damage to the cell envelope. This disruption leads to leakage of cellular contents and ultimately results in bacterial cell death. As a result, bacterial attachment and subsequent biofilm formation are inhibited. This physical antibacterial mechanism is commonly referred to as the “nano-knife” effect (Chen et al., 2024).

Furthermore, several studies have shown that oxidized carbon nanotubes significantly alter microbial surface interactions. Nanoscale engineering has emerged as a promising approach for inhibiting bacterial adhesion and biofilm formation by clinically important pathogens, including *S. aureus* and *P. aeruginosa* (Kang et al., 2007)*.*

Similarly, combining CNTs with antimicrobial agents, such as metals like Cu or metal oxides, significantly increased anti-biofilm activity; these are called metal-functionalized CNTs. The antimicrobial activity of metal-functionalized CNTs arises from the synergistic interaction between the intrinsic antimicrobial properties of the metal components and the membrane-disrupting effects of CNTs, resulting in enhanced bacterial inactivation and biofilm inhibition, as listed in Table 2. Cu is commonly incorporated into CNT-based materials because of its proven antimicrobial activity and clinical relevance (Liu et al., 2009).

**Table 2:** Applications of Carbon-based nanotubes.

Carbon-based products	Mechanism of biofilm inhibition	Application	References
Single-walled carbon nanotubes	Through a sharp structure, bacterial cells get disrupted	Drug delivery and coating	(Iijima, 1991)
Sigma Aldrich multi-walled carbon nanotubes	Inhibition of adhesion and growth	Medical implants	(De Volder et al., 2013)
Carboxyl group functionalized multi-walled carbon nanotubes	Prevention of microbial attachment	Biomedical coating	(Müller et al., 2009)
Double-walled carbon nanotubes	Better penetration into the biofilm matrix	Drug delivery system	(Gabriel et al., 2005)
CNT and antibiotic coating	Sustained drug release inhibition	Orthopedics implants	(Saral et al., 2009)
CNT polymer composites	Prevent biofilm by reducing adhesion and kills the bacteria	Water treatments	(Schwickert et al., 2007)
CNT paste	Forms antimicrobial thin films	Surface coating	(Hinds et al., 2004)

## 4 Anti-microbial properties of Cu-CNTs

Cu plays a significant role in inhibiting biofilm formation due to its antimicrobial properties. One of its key advantages is its broad-spectrum antibacterial activity against both Gram-positive and Gram-negative bacteria. Cu exerts its bactericidal effect primarily through a contact-killing mechanism, whereby direct interaction between bacterial cells and the Cu surface rapidly disrupts the cell membrane, compromises membrane integrity, and ultimately leads to cell death. Cu surfaces continuously release Cu^2+^ ions, which penetrate bacterial cells and disrupt essential cellular processes, including enzymatic activity, protein function, and DNA replication, ultimately leading to bacterial cell death. At controlled concentrations, Cu exhibits potent antimicrobial activity while maintaining acceptable biocompatibility for several biomedical applications.

**Fig. 2:**
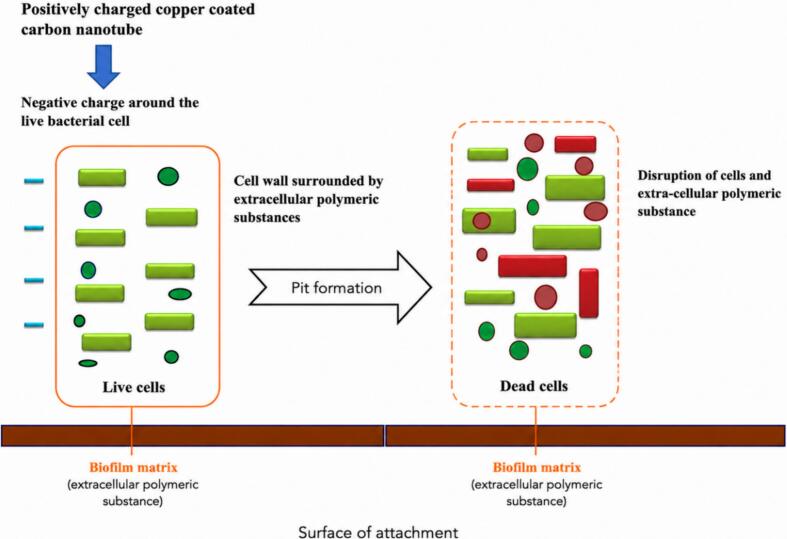
Mechanism of Cu-CNTs against bacterial cells.

Bacterial adhesion to surfaces and quorum sensing are two critical processes involved in biofilm development (Subramani et al., 2026; Venkatesan et al., 2026). Cu has been shown to interfere with both mechanisms through its potent antimicrobial activity, reducing bacterial viability prior to surface attachment and subsequent biofilm establishment, as illustrated in Fig. 2. Hence, Cu-CNTs have attracted considerable attention for biomedical applications, including medical device coatings, implant surfaces, and wound dressings, where prevention of microbial colonization and biofilm formation is essential (Grass et al., 2011).

Cu can be incorporated into carbon nanotubes, both single-walled and multi-walled. But the use of Cu in multi-walled showed better results (Gomes et al., 2025). The incorporation of Cu damaged the cell membrane, reduced bacterial metabolic activity, and disrupted biofilm integrity (Table 3), leading to a decrease in biofilm thickness (Vincent et al., 2018).

**Table 3:** Antimicrobial activity of Cu against microorganisms associated with different anatomical sites.

Application area	Representative microorganisms	Antimicrobial action of Cu	Reported antimicrobial effect	References
Wound dressings and skin-contact surfaces	*Staphylococcus aureus*	Release of Cu^2+^ ions causes membrane damage, oxidative stress, and protein dysfunction	Reduction of bacterial burden and prevention of biofilm formation in wounds	(Grass et al., 2011)
Dental materials and oral-contact surfaces	*Streptococcus mutans*	Inhibition of bacterial adhesion and disruption of biofilm establishment	Reduced plaque formation and bacterial colonization	(Verran and Whitehead, 2005)
Urinary catheter coatings	*Escherichia coli, Klebsiella pneumoniae*	Prevention of bacterial attachment and interference with biofilm development	Reduced catheter-associated biofilm formation	(Venkatesan et al., 2014)
Blood-contact medical devices	*Staphylococcus epidermidis, S. aureus*	Cu-mediated protein dysfunction and membrane disruption	Reduced microbial survival on device surfaces	(Lemire et al., 2013)
Orthopedic implants and implant coatings	*S. aureus, Pseudomonas aeruginosa*	Inhibition of bacterial adhesion and biofilm maturation	Reduced implant-associated infections and biofilm accumulation	(Vincent et al., 2018)
Cu- CNT surfaces	*S. aureus, P. aeruginosa*	Combined membrane disruption, ROS generation, and inhibition of bacterial adhesion	Significant reduction in biofilm formation on coated surfaces	(Bowden et al., 2025)

## 5 CNT effects on *S. aureus* biofilm formation

CNT surfaces significantly reduce bacterial adhesion and inhibit subsequent biofilm development by disrupting bacterial membrane integrity and impairing cellular functions. Cu-coated CNT surfaces significantly reduced *S. aureus* adhesion and biofilm formation compared with unmodified surfaces. The Cu-coated CNT surfaces achieved up to a 99.9% reduction in *S. aureus* biofilm within 12 h. The nanotube structure helps create physical barriers that inhibit bacterial adherence and settlement. These compounds damage bacterial cell membranes and reduce metabolic activity. The resulting decrease in metabolic activity compromises biofilm development, leading to reduced biofilm thickness and structural integrity. CNTs further contribute through direct physical interactions with bacterial cells, causing membrane damage via contact-mediated killing mechanisms. The incorporation of Cu enhances the system's antimicrobial efficacy, while the high surface area of CNTs increases bacterial exposure and mechanical stress. Consequently, the synergistic interaction between Cu and CNTs produces substantially greater antibiofilm activity than Cu alone (Bowden et al., 2025).

## 6 CNT effects on *P. aeruginosa* biofilm formation

The Cu-CNT surfaces exhibited strong antibiofilm activity against *P. aeruginosa* by effectively preventing initial bacterial adhesion, a critical step in biofilm development. These surfaces induced a 6.9-log reduction in adherent *P. aeruginosa* cells, demonstrating substantial inhibition of biofilm formation. Furthermore, CNT-based composite materials reduced biofilm biomass by more than 50% relative to control surfaces. The enhanced antibiofilm performance is attributed to the combined effects of Cu-mediated antimicrobial activity and the unique nanotopography of CNTs, which limit bacterial attachment and colony establishment. Consequently, Cu-modified CNT surfaces showed minimal bacterial adhesion and effectively suppressed biofilm formation compared with conventional material surfaces. Cu-CNT surfaces exhibited strong antibiofilm activity against *P. aeruginosa* by reducing bacterial adhesion, suppressing colony establishment, decreasing EPS production, and disrupting membrane integrity. Collectively, these effects substantially impaired biofilm development compared with conventional surfaces (Hans et al., 2013).

## 7 Biocompatibility and safety considerations

Low Cu ion release and reduced toxicity were observed in Cu-CNT surfaces, indicating effective antibacterial activity with minimal harmful effects on surrounding tissues. Cu-CNT surfaces exhibit potent antimicrobial activity while releasing relatively low concentrations (6.2 ppm) of Cu ions. Their antibacterial effect is primarily mediated through direct surface contact, thereby minimizing systemic exposure and reducing the risk of toxicity. Consequently, these coatings have attracted considerable interest for use in medical devices and implantable materials, where prevention of bacterial colonization is essential without adversely affecting surrounding tissues. Nanostructured CNT surfaces inhibit bacterial growth without requiring toxic chemical concentrations (Algadi et al., 2024).

## 8 Application

The CNT-based antibiofilm materials have important applications in biomedical and environmental fields. The CNT coatings have been widely studied for medical devices such as catheters, implants, and prosthetics, as they prevent microbial adhesion and biofilm formation. The CNTs form aggregated pores with mesopore-sized dimensions, providing a large external surface area that can immobilize large biological contaminants, including bacteria and viruses. In addition, carbon nanotubes also participate in wastewater treatment. The CNT composites are used in water purification systems to prevent biofilm accumulation on the membrane and enhance the filtration efficiency (Raphel et al., 2016).

## 9 Challenges, limitations, and knowledge gaps

Despite their promising antimicrobial and antibiofilm properties, CNT-based materials present several limitations that may hinder their widespread application. Their interactions with biological systems remain an important area of investigation, as CNTs can induce the generation of ROS, leading to oxidative stress, inflammatory responses, and cellular damage under certain conditions. The extent of these effects is influenced by multiple factors, including CNT dimensions, surface chemistry, functionalization, concentration, and exposure duration. Consequently, the long-term biocompatibility and safety of CNTs, particularly for permanent or prolonged biomedical applications, remain subjects of ongoing investigation.

Another major challenge is the lack of standardized protocols for CNT synthesis, functionalization, and purification. Variations in production methods can lead to significant heterogeneity in the physicochemical properties of CNTs, resulting in inconsistencies in their biological performance and toxicological profiles. Furthermore, the environmental fate of CNTs raises additional concerns. Following their release into the environment, CNTs may become bioavailable to a wide range of organisms, potentially resulting in bioaccumulation and trophic transfer through food webs, thereby posing risks to ecosystem health. In addition, the antimicrobial activity of CNTs may adversely affect beneficial microbial communities involved in wastewater treatment processes, disrupting essential metabolic functions and reducing the efficiency of sewage management systems (Springer et al., 2017).

## 10 Future perspective

Future research should focus on developing biocompatible CNTs with reduced toxicity and improved safety for biomedical applications. The modification of CNTs with chemical groups or biomolecules can enhance their antimicrobial efficiency. There is now growing interest in hybrid nanomaterials, such as CNTs combined with metals (Cu, silver) or antimicrobial peptides, which enhance antibiofilm activity through synergy. Further clinical trials are needed to translate laboratory findings into real-world medical applications (Mou et al., 2010).

## 11 Conclusion

Cu-CNTs have demonstrated superior antibiofilm activity compared with uncoated CNTs, highlighting the advantages of combining nanostructured surfaces with antimicrobial metals. The enhanced efficacy of Cu-CNTs arises from the synergistic interaction between the physical membrane-disrupting properties of CNTs and the antimicrobial activity of Cu. This dual-action mechanism effectively inhibits bacterial adhesion, compromises cell membrane integrity, interferes with quorum-sensing pathways, and reduces EPS production, all of which are critical processes in biofilm development and persistence. Consequently, Cu-coated surfaces not only prevent initial bacterial attachment but also impair biofilm maturation and stability.

These findings underscore the potential of Cu-CNTs for preventing biofilm-associated infections, particularly in clinical settings where biofilms contribute to chronic, device-related, and antibiotic-resistant infections. Furthermore, the controlled release of Cu ions, coupled with the predominance of contact-mediated antimicrobial activity, may offer a safer and more sustainable approach for biomedical applications. Potential applications include medical implants, antimicrobial coatings, wound-care materials, and hospital-contact surfaces. Overall, the incorporation of Cu significantly enhances the antibiofilm performance of CNTs, positioning Cu-CNTs as a promising strategy for biofilm control. Future research should focus on in vitro validation, comprehensive biocompatibility assessments, long-term safety evaluations, and scalable manufacturing approaches to facilitate their translation into clinical and healthcare settings.

## CRediT authorship contribution statement

**Abiraami Kalaiselvi Marimuthu:** Writing – original draft, Data curation. **Rithik Roshini Gopi:** Writing – original draft, Data curation. **Jahnavi Padmavathi Sridhar:** Writing – original draft, Data curation. **Kalaivani Ravi:** Writing – original draft, Data curation. **Aditi Roy:** Writing – review & editing, Data curation. **Sudha Ramaiah:** Writing – review & editing, Funding acquisition, Conceptualization. **Anand Anbarasu:** Writing – review & editing, Supervision, Project administration, Conceptualization.

## Ethical approval

This does not apply to our study.

## Declaration of generative AI and AI-assisted technologies in the writing process

During the preparation of this work, the author used ChatGPT and Grammarly in order to improve the readability of the article. After using this tool/service, the author reviewed and edited the content as needed and takes full responsibility for the content of the publication.

## Funding

The author(s) gratefully acknowledge the Indian Council of Medical Research (ICMR), the Government of India agency, for the research grant (IRIS ID: 2021-11889). The funding does not cover article publishing charges (APC).

## Declaration of competing interest

The authors declare that they have no known competing financial interests or personal relationships that could have appeared to influence the work reported in this paper.

## Data Availability

No data was used for the research described in the article.
